# Periodic Catatonia in a Veteran With Post-traumatic Stress Disorder (PTSD) and Traumatic Brain Injury (TBI)

**DOI:** 10.7759/cureus.114754

**Published:** 2026-08-18

**Authors:** Catherine Paciotti, Katarina Bojkovic, Greg Sullivan, Adam Fusick

**Affiliations:** 1 Psychiatry and Behavioral Neurosciences, University of South Florida Health, Tampa, USA; 2 Psychiatry and Behavioral Neurosciences, University of South Florida, Tampa, USA; 3 Mental Health and Behavioral Sciences, James A. Haley Veterans Hospital, Tampa, USA; 4 Psychiatry and Behavioral Neurosciences, University of South Florida Morsani College of Medicine, Tampa, USA

**Keywords:** catatonia, periodic catatonia, psychopharmacology, ptsd, tbi

## Abstract

Periodic catatonia is a rare subtype of catatonia characterized by a variety of neuropsychiatric symptoms that follow a cyclical pattern. These episodes typically involve alternating phases of catatonic symptoms separated by periods of remission. Despite this condition being rare, it is still considered a medical emergency and usually requires inpatient care for assessment and treatment. Unfortunately, managing periodic catatonia can be challenging, largely because its underlying biological causes are not well understood and appear to be less clearly defined as compared to other forms of catatonia. This case report features a 54-year-old male U.S. veteran diagnosed with post-traumatic stress disorder (PTSD) and traumatic brain injury (TBI) after military service who has suffered multiple episodes of catatonia symptoms since being diagnosed with PTSD 21 years before presentation. He presented to the hospital for catatonic symptoms, where the patient was treated with IV lorazepam. Despite early clinical improvement after 24 hours, the patient became highly sedated. Chart review revealed similarities to prior catatonic episodes requiring hospitalization, including normal medical workups. This report is the first to describe a case of periodic catatonia in a patient with PTSD and TBI. Further, the ideological complexity of periodic catatonia is elucidated in order to provide clinical insights into its management.

## Introduction

Once thought to be a subtype of schizophrenia, catatonia is now recognized as a distinct entity associated with both psychiatric and medical conditions [1]. Despite more recent understanding of the syndrome, diagnosing catatonia can still present a clinical challenge. Although various validated clinical scales exist to aid in diagnosis (e.g., Bush-Francis Catatonia Rating Scale (BFCRS), Northoff Catatonia Rating Scale), many features of catatonia--such as immobility, stupor, and autonomic instability--can also be seen in a wide variety of other conditions, including psychiatric disorders, stroke, delirium, functional neurologic disorder, akinetic mutism, and abulia, making catatonia a frequent subject of “medical mimicry” [1,2].

Further complicating the clinical picture, catatonia can be further divided into subtypes, one of which is periodic catatonia (PC). PC is generally defined as a cyclic, relapsing form of catatonia in which patients remain asymptomatic outside of acute episodes [3,4]. The prevalence of PC remains disputed, with some sources citing up to 14% of inpatients with schizophrenia as meeting criteria for PC, while others describe PC as much less common [3,5]. PC is thought to have a strong genetic component, inherited in an autosomal dominant manner with an approximate 27% likelihood in first-degree relatives [6].

Treatment in PC must address both the acute and long-term phases. In the short term, PC often responds to IV benzodiazepines, which are a standard in catatonia management; however, patients may relapse within as little as 48 hours, often requiring additional psychopharmacologic strategies such as amantadine or clozapine [4,5]. The British Association for Psychopharmacology notes that lithium is the most frequently used medication for long-term management of PC, although other groups have reported success with lamotrigine and carbamazepine [1].

Recent research has also implicated external factors in the development of catatonia, particularly trauma. Trauma may contribute through physical injury, as in traumatic brain injury (TBI), or through neurobiological changes associated with psychological trauma [7-9]. Other studies describe post-traumatic stress disorder (PTSD)-related catatonia after surviving traumatic experiences such as false imprisonment and sexual assault [10]. Notably, Dunn et al. state: “It is essential that patients who present with symptoms of catatonia be screened for trauma” [10].

The following case report details, a patient with a prolonged history of periodic catatonia beginning after a traumatic event that led to a diagnosis of PTSD and TBI.

## Case presentation

A 54-year-old male U.S. veteran with a past psychiatric history significant for moderate TBI from blast exposure (resulting in loss of consciousness over 30 minutes and disorientation lasting 24 hours) and PTSD related to combat trauma, including a grenade blast and a gunshot wound to the finger, who also has a history of childhood trauma and a relevant family history of catatonia. He presented to the Emergency Department with acute inability to move or speak. On initial evaluation, he was non-reactive to pain and exhibited classic features of catatonia, including mutism that soon progressed to echolalia, fixed gaze with non-reactive staring, posturing, and asymmetric rigidity. His Bush-Francis Catatonia Rating Scale (BFCRS) score was 12 (3 points for echolalia, 3 for fixed gaze, 3 for posturing, and 3 for rigidity), and he was given a 2 mg IV lorazepam challenge, to which he responded well, with the BFCRS decreasing to 2 (1 point for staring and 1 for rigidity). The patient was subsequently admitted for management of catatonia.

On subsequent psychiatric assessments, the patient's catatonic symptoms appeared well controlled on 2 mg IV lorazepam every six hours for nearly 24 hours until he became excessively sedated, prompting a reduction to 2 mg IV every eight hours. Collateral information from his wife revealed that the patient has had catatonic episodes beginning at age 33 (see Table 1 for a complete time course of his history). These episodes presented with combinations of echolalia, posturing, immobility, staring, rigidity, and, at times, autonomic instability. After emerging from these episodes, he experienced amnesia regarding events shortly before and after. Similar to his current episode, historical episodes were preceded by worsening PTSD symptoms and self-isolation. Notably, some brief catatonic symptoms resolved without intervention, although more severe episodes required medical attention. Historically, when hospitalized, he responded well to lorazepam challenges, consistent with the current presentation. Chart review also revealed that prior EEGs did not show seizure activity, even when performed during catatonic episodes. Given concerns for periodic catatonia and recurrent episodes spanning nearly two decades, a risk-benefit discussion was held, and lamotrigine was initiated at 25 mg orally.

**Table 1:** Overview of patient's psychiatric history. PTSD: post-traumatic stress disorder; TBI: traumatic brain injury.

Age	Event
24	Began service in the United States Marine Corps.
28	Hospitalized in South Carolina due to depression.
32	Deployed to Iraq, TBI and PTSD after blast exposure, sniper injury causing left ring finger amputation and early return home due to trauma.
33	Reported signs of rigidity and posturing, briefly hospitalized.
34	Reported episode of echolalia, immobility and rigidity as well as memory loss following worsening of his PTSD symptoms prompting hospitalization.
35	Discharged from U.S. Marines, hospitalized again for PTSD symptoms and amnesia episodes; only rigidity noted on this admission
36	Hospitalized with symptoms of echolalia, rigidity and autonomic instability following worsening of his PTSD symptoms.
38	Hospitalized with memory loss following exposure to trauma trigger.
40	Hospitalized for catatonia (immobility, echolalia and posturing present). Worsening nightmares reported to precede this admission
43	Hospitalized for worsening PTSD symptoms (flashbacks and derealization)
44	Hospitalized for worsening PTSD symptoms (flashbacks)
48	Hospitalized for worsening PTSD symptoms (flashbacks and derealization)
50	Hospitalized for suicide ideation

Regarding the patient's PTSD symptoms, he described recurrent nightmares of death and being shot at in Iraq. He reported flashbacks three to four times per week during which he re-experienced combat, hearing radio transmissions as if missions were being called in, and seeing dead bodies. He also reported avoiding crowded places and experiencing feelings of detachment and estrangement. He has had episodes of derealization, describing sensations of being outside his body, as well as self-described “blackouts” during which he appeared normal to family but retained no memory afterward. The patient reported that these episodes sometimes occurred concurrently with his catatonic symptoms, although not exclusively. Conversely, his catatonic episodes always presented with periods of amnesia. Prior to admission, he was taking venlafaxine 225 mg orally every morning and terazosin 5 mg orally twice daily for PTSD management.

On hospital day four, after transitioning IV lorazepam to oral dosing, the patient continued to show no signs of catatonia, voiced no acute safety concerns, and no longer met criteria for hospitalization. He was discharged home with a scheduled lorazepam taper (decrease from 2 mg every eight hours to 1 mg every six hours for the first week) while continuing venlafaxine, terazosin, and newly initiated lamotrigine, with the plan to increase from 25 mg to 50 mg after two weeks. His spouse was educated on medication changes, signs of relapse, and return precautions. Close outpatient follow-up was arranged within one week of discharge. 

## Discussion

This case highlights the atypical presentation of catatonia in an individual with a complex trauma history. The patient exhibited relapsing symptoms consistent with PC, but the temporal relationship of these symptoms presenting after worsening of his PTSD symptoms makes this case the first known reported PC case attributable to a complicated trauma history.

Comparing this case to the literature reveals similarities to other reports of the rare diagnosis of PC. Both Hervey et al. and Chen et al., in separate accounts, describe patients with PC who were treated with lorazepam and initially improved, but experienced relapse soon after, necessitating additional psychiatric medication [4,5]. Another case report details a young adult male with PC (and schizophrenia) who underwent a two-week lorazepam treatment course (up to 10 mg/day) without symptom resolution, with a BFCRS score of 21, eventually requiring a combination of haloperidol and diphenhydramine [11]. In this case, after the initial improvement of his symptoms with lorazepam, lamotrigine 25 mg orally was initiated for mood stabilization and the potential long-term benefit of decreasing the frequency and severity of PC episodes.

Of note, the patient's psychiatric history is significant for both TBI and PTSD, both of which contribute to his clinical picture. Several case reports have attributed catatonia to TBI [8,12]. One such report describes a 24-year-old male who suffered a TBI in a motorcycle accident and subsequently presented with suspected catatonia, despite no prior psychiatric history. His symptoms improved with an initial lorazepam challenge, and when lorazepam was discontinued, his catatonic symptoms worsened, though the exact timeline was not specified [12]. Another case, reported in 2024 by Berthelot and colleagues, described the case of a 20-year-old male who presented with catatonic symptoms shortly after a TBI sustained during a physical assault while incarcerated. This patient improved immediately after a lorazepam challenge [8].

Relatedly, the patient's PTSD history also contributes significantly to his presentation. It has been well established that PTSD can present with dissociation and, in some instances, possible prolonged episodes of amnesia [13]. Given that his PC symptoms presented following worsening of his PTSD symptoms (namely, avoidance, increased flashbacks, and depersonalization), as well as amnesia around these episodes, it is likely that his PTSD is triggering his PC.

As this is the first case report linking PC and PTSD, no other literature exists to which we can compare our patient. However, there are multiple reports affirming the occurrence of PTSD-related catatonia [7,9]. Ahmed et al., for example, describe a 12-year-old patient who presented with catatonic symptoms (mutism, non-ambulation, and enuresis/encopresis) after surviving verbal and physical abuse (of note, this patient was soon thereafter diagnosed with PTSD). A three-day lorazepam treatment course provided minimal benefit, but her symptoms resolved completely after electroconvulsive therapy (ECT) [7]. By comparison, Bonomo et al. describe a middle-aged female patient with PTSD with catatonic features, including waxy flexibility, stupor, and complete mutism. Although this patient failed benzodiazepine therapy (and was not a candidate for ECT), she demonstrated rapid improvement after administration of oral zolpidem [9]. These cases collectively emphasize that treatment for catatonia, and by extension PC, can be multifaceted, often requiring modification of the initial treatment plan until symptoms are appropriately managed.

Still, the underlying pathophysiology of catatonia remains poorly understood, as etiology and presentations can vary widely. A 2024 study evaluating fMRI findings in catatonia sheds some light as to why this syndrome is rather complex. At a neuroanatomical level, Duque et al. noted distinct imaging patterns, depending on whether catatonia was secondary to psychiatric versus medical causes. In psychiatric-related catatonia, the prefrontal cortex (PFC), supplementary motor area (SMA), and ventral premotor cortex demonstrated hyperactivity, whereas hypoactivity in the PFC and SMA was observed in medical-related catatonia [14]. Research from Strasbourg, focusing on biomarkers for PC using arterial spin labeling MRI, found increased regional cerebral blood flow in the left SMA and the left lateral premotor cortex [15]. These overlapping findings may reflect shared symptomatology across catatonia subtypes.

Furthermore, one of the most significant distinctions between PC and other types of catatonias is the need for long-term psychopharmacologic intervention to reduce relapse severity and duration. Although the mechanism of PC remains unclear, other forms of catatonia are associated with down-regulation of gamma-aminobutyric acid type A (GABA-A) receptors and excessive glutamatergic neurotransmission [1]. Given that lamotrigine, lithium, and carbamazepine have been identified as effective maintenance treatments for PC, it is worth considering these agents' neurochemical actions in the context of catatonic pathophysiology [1]. Lamotrigine has been shown to increase the release of GABA and improve mood stabilization in patients with a diagnosis of TBI or PTSD, making it a particularly relevant medication in our patient's case [16,17]. Carbamazepine exhibits modest GABAergic and antiglutamatergic activity, while lithium enhances GABA uptake [18,19]. Understanding catatonic neurobiology may help clinicians make more informed decisions when selecting long-term treatments for PC.

Finally, treating catatonia effectively requires addressing not only the immediate motor and behavioral symptoms, but also the underlying causes that contribute to its onset [1]. PTSD and TBI are recognized as possible etiologies of catatonia and targeting trauma-related symptoms through appropriate psychiatric and therapeutic treatment can generally lead to better control and reduction of catatonic episodes. In this case, although several psychiatric medications were considered, lamotrigine was selected due to its demonstrated success in the existing literature as an adjunctive agent for management of behavioral symptoms of TBI, as well as symptoms of PTSD [17,20]. Prioritizing comprehensive evaluation and treatment, therefore, improves both symptom relief and overall patient outcomes.

## Conclusions

This case underscores the complexity of catatonia in the context of comorbid PTSD and TBI. The patient's recurrent, episodic presentations over two decades, consistent responsiveness to lorazepam, and symptom-free intervals align most closely with periodic catatonia (PC). This report reinforces the importance of screening for trauma in patients with atypical or recurrent catatonic features and recognizing that differing pathophysiologic mechanisms across catatonia subtypes may necessitate distinct pharmacologic approaches, including the use of mood stabilizers for long-term benefit.
